# Colostomy Takedown: Ischemic Complication following Anorectal Malformation Surgery

**DOI:** 10.1155/2021/8870631

**Published:** 2021-01-12

**Authors:** Wendy Jo Svetanoff, Justin Sobrino, Grace S. Mitchell, Rebecca M. Rentea

**Affiliations:** ^1^Comprehensive Colorectal Center, Department of Surgery, Children's Mercy Kansas City, Kansas City, MO 64108, USA; ^2^Department of Radiology, Children's Mercy Kansas City, Kansas City, MO 64108, USA

## Abstract

**Introduction:**

Anorectal malformations (ARM) are complex disorders that often require staged reconstructions. We present a case and imaging findings of a child who developed issues following colostomy closure due to segmental colonic ischemia. *Case Presentation*. A 3-year-old female with Currarino syndrome presented with abdominal distention, blood-flecked stools, and prolonged cecostomy flush time. For her anorectal malformation, a colostomy was initially placed. A new colostomy was created at posterior sagittal anorectoplasty (PSARP) to allow the distal rectum to reach the anus without tension. Differentials for her presenting symptoms included a mislocation of the anus, stenosis at the anoplasty site, stricture within the colon, or sacral mass from Currarino syndrome, causing obstructive symptoms. Workup at our hospital included an anorectal exam under anesthesia (EUA), which showed a well-located anus with without stenosis at the anoplasty site, and an antegrade contrast study revealed a featureless descending colon with a 3-4 mm stricture in the distal transverse colon at the site of the previous colostomy, without an obstructing presacral mass. To alleviate this obstruction, the child underwent removal of the chronically ischemic descending colon and a redo-PSARP, where the distal transverse colon was brought down to the anus. She is now able to successfully perform antegrade flushes.

**Conclusion:**

Patients who have had prior surgeries for ARM repair are at a higher risk of complications, including strictures or ischemic complications at areas of previous surgery or colostomy placement. A thorough preoperative workup, including contrast studies, can alert the surgeon to these potential pitfalls.

## 1. Introduction

Anorectal malformations (ARM) affect 1 in 5000 children [1], with approximately 50% requiring the creation of a sigmoid or descending colostomy before definitive surgical reconstruction. While a colostomy is safe and often necessary, the rate of complications ranges from 28to 74% [1, 2]; problems with prolapse, anastomotic breakdown, strictures, and intestinal obstruction can occur and often require reoperation [1–4]. In children with ARM who are struggling with fecal soiling or antegrade enema access issues, the performance of a contrast enema or antegrade contrast study can identify if the previous colostomy or closure site is contributing to the current problem [5, 6]. We present the case and radiologic images of a child who presented with abdominal distention and fecal soiling despite antegrade enema access route and was found to have a functional obstruction caused by a long segment ischemic stricture in the descending colon.

## 2. Case Presentation

A 3-year-old female was diagnosed one week after birth with a rectovestibular fistula and presacral mass. Her initial surgery consisted of an anorectal exam under anesthesia (EUA) and diverting divided sigmoid colostomy. Other congenital defects included an atrial septal defect, bilateral vesicoureteral reflux, left hemi-sacral agenesis, tethered cord, anterior myelomeningocele, and didelphys uterus. At 3 months of age, she underwent an intrapelvic myelomeningocele repair and tethered cord release. At 7 months of age, a posterior sagittal anorectoplasty (PSARP), takedown of the sigmoid colostomy, and creation of a transverse colostomy were performed as the rectum was unable to reach the anoplasty site with the current colostomy still in place. Following colostomy reversal, the patient developed episodes of bloody stools, severe constipation requiring senna and lactulose, as well as intermittent saline irrigations. At 2 years of age, given the difficulty with various laxative regimens, a cecostomy tube was placed.

**Figure 1:**
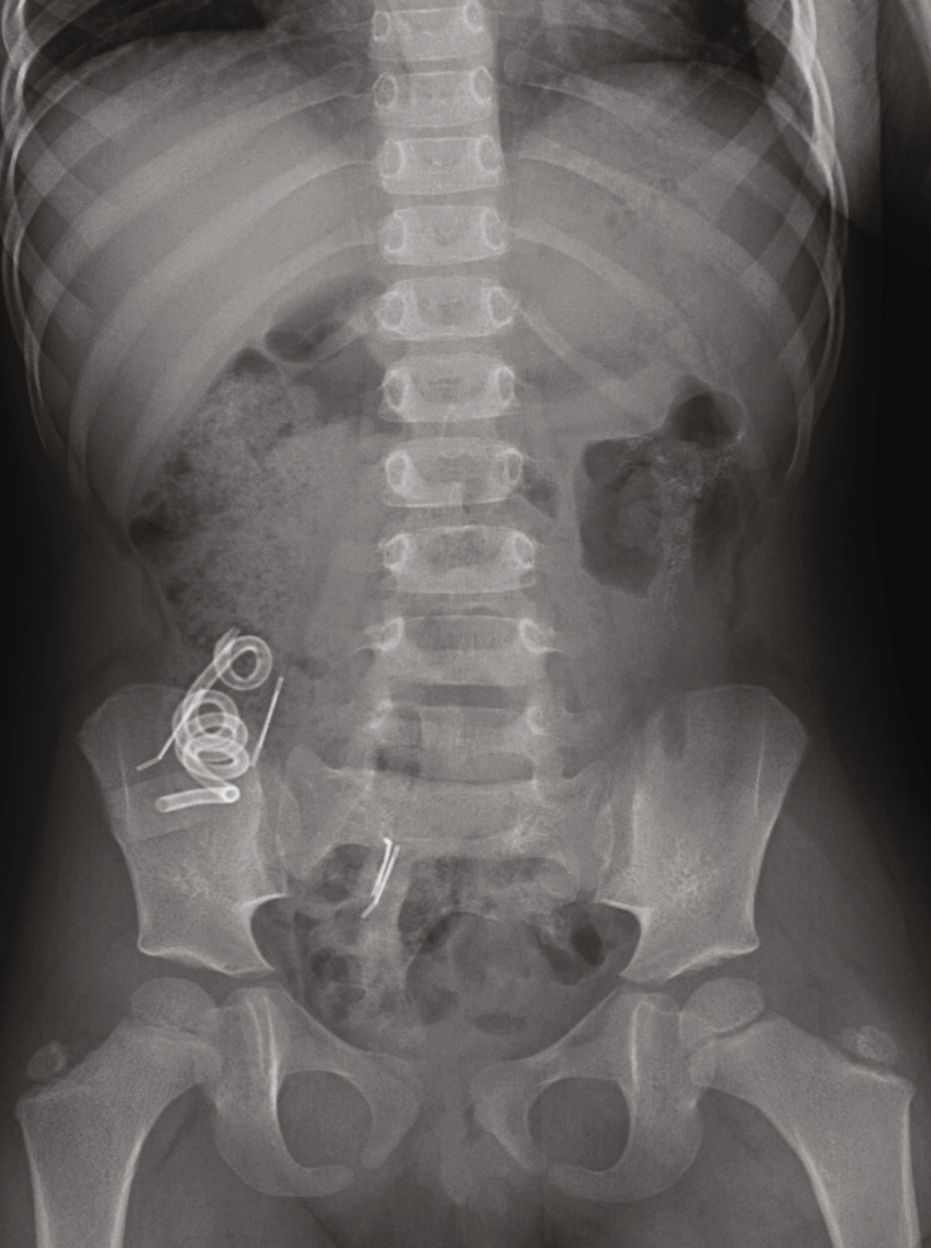
Supine frontal radiograph of the abdomen demonstrates the cecostomy catheter and surgical hardware overlying the right lower quadrant. Stool is seen throughout the colon. A dysmorphic sacrum is seen curving to the right, consistent with a known diagnosis of Currarino triad.

**Figure 2:**
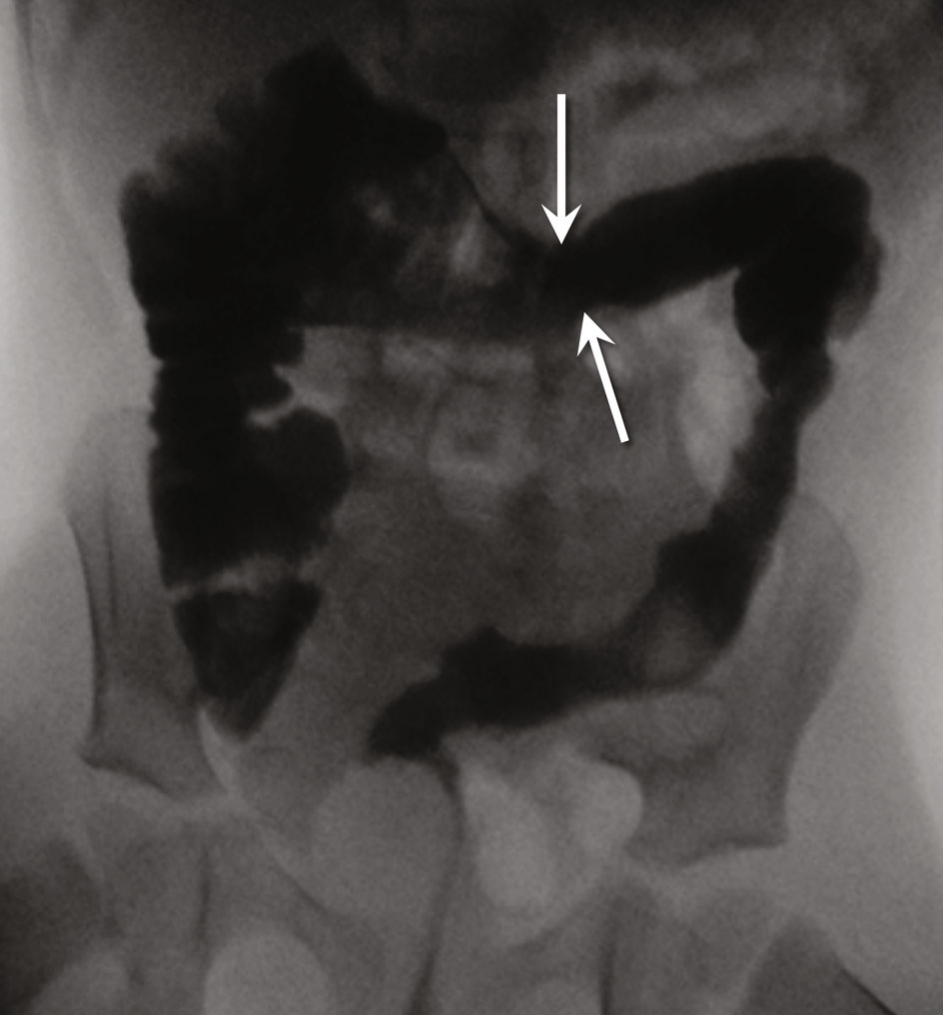
Frontal image from *retrograde* water-soluble contrast enema demonstrates narrowing of the left colon, with loss of the typical haustral markings. A transition point is identified in the mid-distal transverse colon (arrows). The right colon is normal.

The patient presented to our institution at 3 years of age with abdominal bloating, a history of blood-flecked stools, and prolonged cecostomy flush evacuation. Her flush consisted of 400 mL of saline and 25 mL of glycerin. The differential diagnosis for her symptoms included a mislocated anus from her previous PSARP, a stricture at the anoplasty site, a stricture, or other anatomic abnormality of the colon (such as a twist of the pull-through), or a presacral mass obstructing the colon. Rectal EUA revealed a well-located anus and rectal vault without stricture at the anoplasty site. An abdominal radiograph (Figure 1) and retrograde and antegrade contrast studies were obtained to further evaluate an anatomic abnormality (Figure 2).

**Figure 3:**
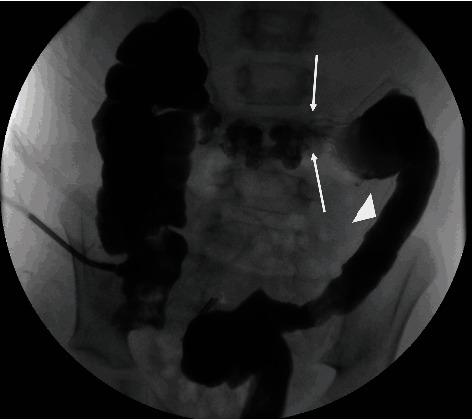
Frontal image from *antegrade* water-soluble contrast study through the cecostomy demonstrates narrowing of the left colon, with loss of the haustral markings (small arrow). A transition point is identified in the distal transverse colon (arrows). A more proximal transverse colon has normal haustral markings but is incompletely distended.

Antegrade contrast study through the cecostomy (to evaluate the proximal colon) demonstrated a focal transition point measuring 3-4 mm in the distal transverse colon and a small, featureless descending colon (Figure 3).

**Figure 4:**
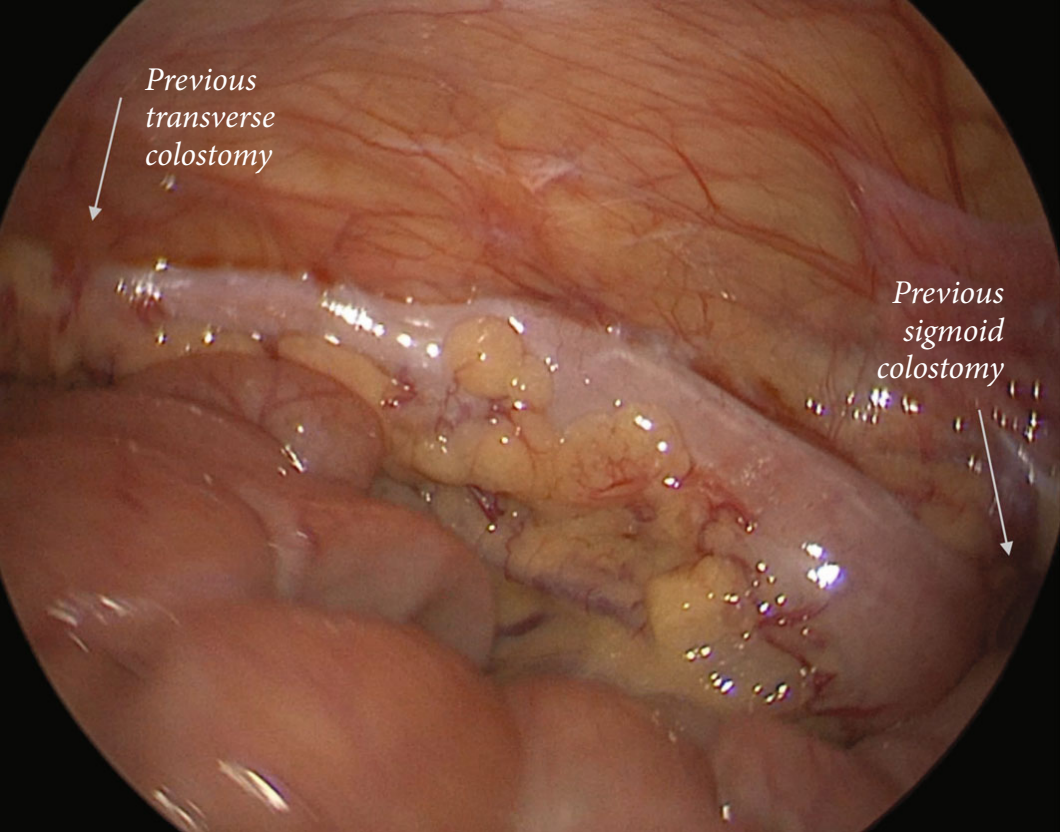
Intraoperative view of the descending colon. Upon inspection, the descending colon was narrow and featureless. The sites of the previous colostomies are highlighted.

To further delineate the pathology and allow for ascending colon decompression, the patient underwent an exploratory laparoscopy. The anastomosis was patent; however, the descending colon appeared abnormally thick-walled, was narrow in caliber, and featureless, with a thin mesentery from the previous transverse colostomy site to the peritoneal reflection and significant scarring at the level of the previous sigmoid colostomy and rectum (Figure 4). Given the focal narrowing in the distal transverse colon, a diverting ileostomy was performed to allow ascending colonic decompression.

**Figure 5:**
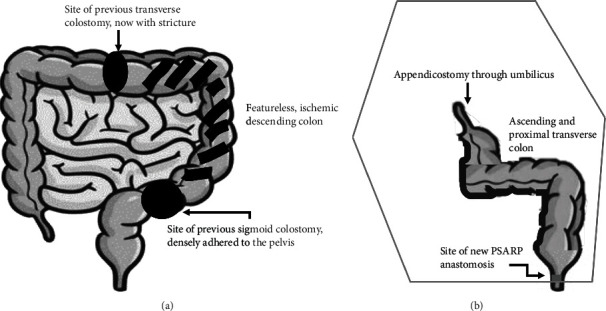
(a) Anatomy of the patient's colon prior to redo-PSARP, indicating the sites of her previous colostomies and the ischemic descending colon. (b) Final anatomic configuration after colon resection and redo-PSARP. Her neoappendicostomy is anastomosed to her umbilicus, while the ascending and proximal transverse colon were derotated to allow for a new coloanal anastomosis.

The patient subsequently underwent excision of the descending colon to include the previous transverse and sigmoid colostomy sites, a redo PSARP, and neoappendicectomy placement. Removal of the colon from the transverse to the sigmoid colostomy site was performed due to the focal narrowing at the transverse colostomy site and the descending colon's ischemic nature, which we were concerned would act as an obstruction point from lack of motility if it remained in place. This was confirmed with the use of indocyanine green fluorescence angiography (SPY), which revealed poor perfusion to the descending colon. The area of the previous sigmoid colostomy was densely adhered to the pelvis with unclear mesentery. After complete mobilization of the colon through the laparotomy incision, SPY technology was utilized to confirm where the blood supply attenuated on the colon. Although the anus was free of stricture, the colon remaining in the pelvis was <5 cm; therefore, a colorectal anastomosis was unable to be performed and a redo PSARP was performed. The patient was placed in lithotomy, and a lone-star retractor was placed to facilitate the anorectoplasty. Multiple sutures were placed through the mucosa of the rectum; circumferential dissection of the distal rectum was performed. Derotation of the colon at the level of adequate blood supply as seen on SPY was performed; the colon was pulled through the anus, ensuring correct orientation. The mucosa was secured to the skin using interrupted sutures (Figure 5). The anastomosis was able to accommodate a 17-18 Hegar dilator easily. Confirmation of colonic viability was performed using SPY. The resected colon had diffuse submucosal edema with focal areas of mucosal and submucosal hemorrhagic necrosis and submucosal fibrosis on pathologic review. The ileostomy was closed a few weeks later.

Currently, the child is in a 1-year post ileostomy closure and redo-PSARP and remains clean for stool with morning flushes with the aid of 2 mg of Imodium in the morning and 4 mg in the evening. Genetic testing has since been performed; both the patient and parent were found to be positive for a pathogenic MNX-1 variant that is consistent with Currarino syndrome.

## 3. Discussion

We present a child who experienced abdominal bloating during antegrade colonic flushes due to a long ischemic descending colon stricture situated between two previous colostomy sites. From this case, there are three unique issues to highlight. First, the relatively larger size of the proximal colon alerts that an obstruction may be present. Second, while nonspecific, the lack of descending colonic haustra can be due to ischemia and may indicate that this part of the colon may not function properly, acting as a functional obstruction. This child had two colostomies with subsequent closure, which sandwiched an ischemic descending colon (likely from disruption of the intervening colonic arcade). Any child who presents with abdominal bloating issues and soiling may have an issue from a colostomy closure site. This goes along with the third issue that the focal stricture could be why this child was experiencing the bloating and prolonged flush times during cecostomy flushes.

While the creation of a colostomy is necessary for initial fecal diversion in a majority of patients, they are not without risk of complications [1–4]. In one study of patients with intermediate or high ARMs, 32% had mechanical complications, including 7 patients who developed intestinal obstruction [1]. Another study found a 29% morbidity rate after colostomy closure [4]. Ischemia of the colon can present clinically with bloody bowel movements or signs of obstruction. It is important to note that the descending colon's marginal blood supply may have been interrupted during the previous surgery for two colostomies. Radiographically, colonic ischemia can be identified as a stricture with bowel obstruction, a featureless colon with a lack of haustra, and/or changes in the caliber of the colon compared to other intestinal segments [6]. Our diagnosis was confirmed by the histopathological report identifying submucosal edema, hemorrhagic necrosis, and fibrosis—findings consistent with ischemia and stricture [7]. A workup that includes an EUA and both retrograde and antegrade contrast studies, when indicated, can identify anatomic abnormalities causing the obstructive symptoms.

In patients with ARMs, the best chance of obtaining continence is with a successful repair at the first attempt [8]; however, for those with complications, a thorough history, EUA, and review of operative and imaging studies to further identify the anatomy are critical to obtaining successful long-term outcomes [6, 8, 9]. For this child, the initial EUA found an appropriately placed anus without a stricture of the anal canal. Thus, further workup was required, and retrograde and antegrade contrast imaging were able to identify the length of the colonic stricture.

## 4. Conclusion

The diagnosis of colonic ischemia or stricture at a previous colostomy site should be considered in children who have issues with fecal incontinence or abdominal bloating following colostomy closure. While a retrograde contrast enema often is reliable in obtaining the diagnosis, an antegrade enema may help to define any proximal colonic dilation and antegrade obstructions. A high index of suspicion should be maintained in patients with abdominal bloating and fecal incontinence in the setting of a previous colostomy.
